# Child participation during outpatient consultations: a mixed methods study

**DOI:** 10.1007/s00431-024-05566-8

**Published:** 2024-04-19

**Authors:** Maud M. Koenis, Heleen Vroman, Paul L. P. Brand, Christiaan S. van Woerden

**Affiliations:** 1 Department of Pediatrics, Bravis Hospital, Boerhaaveplein 1, 4624 VT Bergen op Zoom, the Netherlands; 2 Department of Science, Bravis Hospital, Bergen op Zoom, the Netherlands; 3 Isala Academy, Department of Medical Education and Faculty Development, Isala Hospital, Zwolle, the Netherlands; 4 Wenckebach Institute for Medical Education, University Medical Centre Groningen, Groningen, the Netherlands

**Keywords:** Child participation, Child-centered care, Pediatric consultations, Children, Participation

## Abstract

Although children wish to be included in their own healthcare, they recognize a gap between their right to be heard and their ability to become involved. Despite adaptation of medical consultation styles which suit a more patient-centered approach, data on the current state of child participation in clinical encounters are missing. We aimed to assess actual child participation in a Dutch pediatric clinic. Children aged 4–18 years visiting a pediatric outpatient clinic for consultation after general practitioner’s referral were included. Sixteen consultations of six pediatricians were recorded and transcribed verbatim. Quantitative measurement included word count and speech turn; conversation analysis with qualitative appraisal provided data on participatory behavior. Quantitative child participation equaled parent participation in turns (28% vs 29%, respectively), but remained limited in words (relative contribution 11% for child, 23% for parent and 66% for pediatrician). Children spoke on average six words per speech turn. Child age correlated positively with participation in words (*p* = 0.022, *r* = 0.566) and turns (*p* =  < 0.001, *r* = 0.746). Children were mostly involved during social history taking, introduction, and physical examination but did not actively speak during the decision-making process. Children took an active role by instigating talks. Qualitative facilitators included appropriate language and verbal or non-verbal child allocated turns. Adults involved children by asking them questions and verifying their opinions or plans with the child. Teenagers participated most during the entire consultation, while younger children were more likely to lose their focus by the end of the conversation.

*Conclusion*: Despite increased turn taking, children’s verbal participation remains low in pediatric consultations. If pediatricians and parents maintain a triadic conversation style throughout every stage of the medical encounter, child participation may increase.

**Table:** 

**What is Known:** *• Verbal child participation varies between 4 and 17%, measured in turns, words, speech time, or utterances.* *• Child participation is limited to social talk, laughter, and providing medical information.*	
**What is New:** *• Child speech turns equal parental speech turns (28%), but average relative word count remains low (11%).* *• Three percent of the children’s turns are defined a “contributing in decision making, giving their opinion or give consent,” which equals three turns per consultation.*

**Supplementary Information:**

The online version contains supplementary material available at 10.1007/s00431-024-05566-8.

## Introduction

The UN Right of Children to be heard and express their views is intended to promote child participation in various societal areas. Nevertheless, child participation in health care has historically been recognized as limited. In a recent scoping review, the child’s verbal contribution to medical encounters ranged between 4 and 17%, as measured in word count, utterances, or relative speech time, depending on the age of the child [1].

The introduction of patient centered care in the past three decades has focused on power sharing between health care professional and patient. In addition to improving this relational interaction, it has emphasized the importance of understanding the patient’s preferences, needs, and values [2]. Healthcare institutions have deployed initiatives such as family-centered care in ward rounds and outpatient clinics to accommodate medical care according to the needs of children. This may have improved child participation in the encounter. However, for almost 20 years, no study has reported on the degree of child participation in medical consultations. To our knowledge, the potential clinically relevant improvement in child participation as a result of a more patient-centered approach in medical consultations has not been documented to date.

In an interview with the national Ombudsman in the Netherlands, Dutch children recently reported dissatisfaction with their involvement in medical care [3]. They denounced not being listened to by health care professionals and being left out of important decision-making conversations. This suggests ongoing violation of one of the fundamental rights of children and underscores the need for studies to assess the current state of child participation. The results of such studies could further advocate the need for child participation promoting initiatives and its evaluation research.

The aim of the present study was to assess actual child participation in Dutch pediatric outpatient care.

## Methods

### Study design

We assessed videorecorded outpatient consultations between pediatricians, child patients, and their parents or caregivers in a large general hospital in a rural-urban area in the Southwest of the Netherlands. A single camera capturing the three participants was used. Recordings were made between April and July 2022. Children or parents who did not speak Dutch or English, children with psychomotor impairment, communication disorders (including non-verbal autism), acutely ill children, and those requiring emergency consultations were excluded. This study was performed in line with the principles of the Declaration of Helsinki. Approval was granted by the Medical Ethics Committee of Isala Zwolle and the local Research and Ethics Board (METC 220206, date March 03, 2022/No. PAC-2022-019). Six pediatricians agreed to take part in the study. All participants, children, parents, and doctors signed informed consent.

### Data collection

Videos were transcribed verbatim including relevant non-verbal communication according to the transcription system explained by Aronsson and Rundström [4]. A semi-structured interview following a short questionnaire was conducted after the consultations with the child and their parents to assess their experience and satisfaction with the interaction.

### Quantitative measures

We analyzed conversational contribution using word count and turn taking (number of speech turns taken by each participant, i.e., what one says between two moments of silence) to study control of the conversation [5]. Non-verbal communication was noted as far as it was relevant for understanding intonation, switching pause or silences. Instigated talks by children were counted and categorized per consultation, adapted from Jenkins et al. [6]. We counted instigated talks, i.e., the child starting a new subject or verbally participating again without having been allocated a turn in the conversation, as adapted from Jenkins et al. [6]. To analyze child participation during various stages of the conversation, we divided the conversation into the following segments: (1) introduction and agenda setting; (2) history taking (exploration of the presenting complaint); (3) social history (school, sports, hobbies); (4) general history (medical/family history, medication, growth, allergies); (5) physical examination; (6) summary, explanation, and decision-making; and (7) closure [7]. This is a more extensive division than the three-segment system as proposed by Tates and Meeuwesen [5] which allowed us to allocate child participation in more detail.

### Qualitative measures

Factors influencing child participation were analyzed in an inductive way by two researchers (MK and CW) using verbatim transcripts and observing video recordings. Based on hypotheses that we generated following inductive analyses, additional deductive studies were performed as appropriate. Signs or patterns that influenced child participation were observed until saturation was reached. For qualitative outcome measurements, thematic analysis was performed.

### Data analysis

Descriptive statistics were performed for quantitative measurements. Continuous variables are presented as mean and standard deviation if normally distributed and with median and interquartile range when non-normally distributed. Associations between variables were assessed using Pearson correlation coefficients. Analyses were performed in SPSS Statistics 28 and Excel software.

## Results

### Quantitative measurements

#### Participant and visit characteristics

Sixteen consultations were videotaped with six pediatricians, 18 children, and 19 parents. The children included in the study were aged between 4 and 17 years (mean 10 years, standard deviation (*SD*) = 3.72). All children were accompanied by one or both parents. The mean duration of the consultation was 31 min and 54 s (*SD* = 8.12) (Table 1).

**Table 1:** Participants and visit characteristics (*n* = 16)

**Child characteristics (*n* = 16)**	
Child male gender (*n*, %)	9 (56%)
Child age in years (mean, SD^a^)	10.1 (3.72)
Age group	
< 12 years (*n*, %)	11 (69%)
> 12 years (*n*, %)	5 (31%)

**Pediatrician characteristics (*n* = 6)**	
Pediatricians female gender	6 (100%)

**Visit characteristics (*n* = 16)**	
Parent presented at visit	
Mother only	11 (69%)
Father only	3 (19%)
Father + siblings	1 (6%)
Both parents	1 (6%)
Reason for consultation	
Cardio-vascular (collapse (*n* = 1))	1
Respiratory (asthma (*n* = 1), allergy (*n* = 1))	2
Gastro-intestinal (abdominal pain (*n* = 3), encopresis (*n* = 1))	4
Skeletal (joint pain (*n* = 1))	1
Endocrine (tall stature (*n* = 1), pubertas praecox (*n* = 1))	2
Neurological (headache (*n* = 1))	1
Infections (lymphadenopathy (*n* = 1))	1
Psycho-social (ADHD (*n* = 1), overweight (*n* = 1))	2
Other (fatigue (*n* = 2))	2
Duration of consultation in minutes (mean, SD^a^)	31.9 (8.12)

^a^Standard deviation

#### Participation during the consultation

**Fig. 1:**
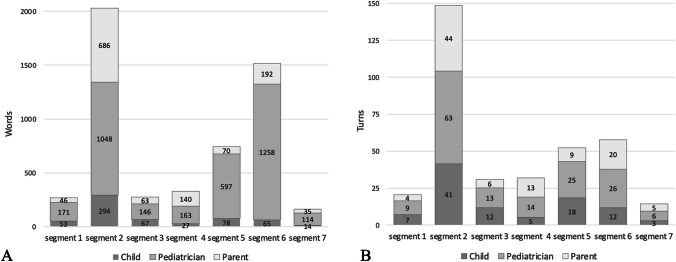
Stacked bar of total word and turn count per person per segment. **A** Words per segment. **B** Turns per segment. Segments: (1) introduction and agenda setting; (2) history taking; (3) social history; (4) general questions; (5) physical examination; (6) summary, explanation, and decision-making; (7) closing

Participation in word count was on average 11.3% (range 2.6–30.9%) for children, 23.3% (range 2.6–49.7%) for parents, and 65.4% (range 41.4–81.7%) for pediatricians. Distribution of turns during the consultations was 27.6% (range 3.4–44.7%) for children, 28.5% (range 10.6–39.1%) for parents, and 43.9% (range 37.4–49.1%) for pediatricians (Table 2). Children spoke on average six words per speech turn. Participation of the child was highest during history taking (segment 2), with an average of 294 words and 41 turns (Fig. 1), which almost equaled the amount of turns of the parents (Fig. 1B). Pediatricians dominated all segments with participation between 52 and 83%. The highest participation of pediatricians was during physical examination (80%) and summary, explanation, and decision-making (83%) (Fig. S1). Child age correlated positively with participation in words (*p* = 0.022, *r* = 0.566) and turns (*p* =  < 0.001, *r* = 0.746) (Table S1; Fig. S2).

**Table 2:** Quantitative outcomes

**Child**	
**Word count (median, 25th–75th percentile)**	425^a^ (200.8–990.3)
**% of words (mean, min-max)**	11.3% (2.6–30.9%)
**Turn count (mean,** ***SD*)**	97 (41,2)
**% of turns (mean, min-max)**	27.6% (3.4–44.7%)

**Pediatrician**	
**Word count (mean,** ***SD*)**	3453 (1370.9)
**% of words (mean, min-max)**	65.4% (41.4–81.7%)
**Turn count (mean,** ***SD*)**	154 (48,6)
**% of turns (mean, min-max)**	43.9% (37.4–49.1%)

Parent	
**Word count (mean,** ***SD*)**	1226 (590,0)
**% of words (mean, min-max)**	23.3% (2.6–49.7%)
**Turn count (mean,** ***SD*)**	100 (42,0)
**% of turns (mean, min-max)**	28.5% (10.6–39.1%)

^a^Mean wordcount children = 598 words (non-normally distributed)

#### Instigated talks

During consultations, children instigated talks between one and 66 times per consultation, consisting 19% of total turns (Fig. S3a). In the majority of instigated talks (73%), the current conversation was joined without direct allocation (Fig. S3b). In the other 27%, a new subject for discussion was started, which occurred in 12 consultations. Pediatricians or parents responded by accepting the new subject (41%), cutting it short (39%), or ignoring it (20%) (Fig. S3c). An example of instigated talk by children is shown in Box 1.

### Qualitative measurements

#### Subject of talk

Thematic analysis showed that more than half of children’s turns (57.6%) were defined as “giving information” (39.9%) and “building relationship/social talk” (17.7%) (Table 3). Only 3.1% of the children’s turns were classified as “contributing to decision-making, give their opinion or give consent,” equaling three turns per consultation.

**Table 3:** Thematic analysis of turns of children

	**Percentage of children’s turns**
1) Giving bio-medical information	39.9%
2) Giving psycho-social/lifestyle information	8.6%
3) Building relationship, social/positive talk	17.7%
4) Asking medical related question	1.3%
5) Asking other/social questions	3.1%
6) Expressions, statements, facts out of context	6.9%
7) Expressing feelings or emotional status	1.8%
8) Contributing to decision-making, giving their opinion, giving consent	3.1%
9) Remaining statements that do not provide information	17.6%

Box 1 Example of instigated talks

**Table:** 

220.	D: I will put this away for now, ((puts away the toy)) so I can examine you. Come sit next to me on the examination bench. You can take off your pants and your T-shirt. Is mom going to help you or can you do that by yourself?
221.	C: Look, I am not, I am not, not wearing an undershirt^a^ ((lifts his T-shirt))
222.	M: You’re not wearing an undershirt^a^, just take off your T-shirt darling ((helps the child taking off his T-shirt))
223.	D: No, you’re not, just take off your T-shirt
224.	C: I am not wearing an undershirt^a^
225.	D: And your pants and your shoes
226.	C: I am not wearing an undershirt^a^
227.	D: You’re not wearing an undershirt^a^ and I know why. It’s because it was going to be very hot today.
228.	C: Yes
229.	D: That is right, isn’t it?

*D* doctor, *M* mother, *C* child, *(())* non-verbal actions

^a^Undershirt = what you wear under clothes to stay warmer

#### Participation of children during consultations

The encounter started with the doctor taking the child and parent(s) out of the waiting room. Children and parents sat on adult-sized chairs on one of the long sides of a rectangular table. The pediatrician was seated opposite of them. A computer screen was placed on the side of the table and turned towards the physician, leaving space for all participants to see each other’s faces. In the beginning of the consultation, children showed active participation with straight posture and eye contact with the pediatrician. Some children leaned forward or moved their chair more closely to the desk. Most pediatricians allocated the first qsuestions to the child, often enquiring the reason for encounter or exploring the present complaint. They reverted to closed or multiple choice questions when the child’s speech was not elicited. Occasionally, they started with a social talk in which children could easily participate. The child’s attention span was often short but could be retained by (non-)verbal facilitators which maintained a triadic conversation style, such as eye contact, triangular seating arrangement, and when parents verified their answers with their child. During physical examination, the interaction was between pediatrician and child exclusively. During the transition from examination bench to desk, children often got left out for summary and plan proposal, when the pediatrician continued the conversation with the parent while the child was still getting dressed. Although in 14 consultations the child’s contribution was classified as “decision-making, giving consent or giving their opinion,” their verbal contribution was minimal during the decision-making segment. The verbal consents in this segement often consisted of confirmatory statements as short as “ok” or “yes” despite the effort of the pediatrician to re-engage the child as shown in the examples in Boxes 2, 3, and 4. In Box 2, the pediatrician rounds up the consultation and sends the child to the receptionist, but notices that he is engaged with a toy on the desk, calls his name, and asks again for consent (“Do you think it is a good idea to round up?”). In another occasion as shown in Box 3, the pediatrician acknowledges the fact that the explanation goes beyond the child’s level of understanding, but promises to come back to the child (“I also want to tell it to you”) and gives a separate explanation at the child’s level of understanding. In the example of Box 4, the pediatrician enquires the child about her desired level of involvement (“What would you like yourself?”) before providing a medical explanation.

Box 2 Example of pediatrician giving a short summary at the end of consultation

**Table:** 

578.	D: That can be the drawback, uhm, let me have a look, I am going to ask if they can give you the ticket at the reception. ((mumbles while typing)) Yes, shall we do it like this?
579.	C: yes
580.	D: Then you are ready here, and you are going to draw blood with mom, this week on a morning, and then I’ll call mom afterwards when it’s all said and done. ((looks at the child who plays with a toy, the balancing bird)) Well, I see a lot of nice ones of those balance birds, also one in blue.
581.	C: 15 euro at bol.com, only at Ali-express they are cheap.
582.	D: Hey “child’s name”, do you think it’s a good idea to round up?
583.	C: Yes
584.	D: then you will search a bit more at home, and I have said that you will draw blood and that you…then I will call mom, yes.
585.	M: Did you hear what she said?
586.	C: ((waves at camera))

*D* doctor, *M* mother, *C* child, *(())* non-verbal actions

Box 3 Example of the pediatrician being aware of low child participation during explanation

**Table:** 

393.	D: Or if there is something like a flu virus, or some allergic stimuli to which you respond less severe currently. It’s a little boring now, isn’t it, because I am going to explain a few things to dad. I also want to tell it to you, hey.
394.	C: Yes
–––	((Father and doctor talk about the medication the child uses))
400.	((Doctor explains something about medication use directed to the father)) “child’s name” shall I explain something to you… ((explains something about allergies and asthma to child))

*D* doctor, *C* child, *(())* non-verbal actions

Box 4 Example of involving the child during decision-making

**Table:** 

164.	D: And what would you like yourself? Would you like to know what it is, or do you think, well, whatever…?
165.	C: I really would like to know what it is.
166.	D: Sounds good to me, then let’s have a look hey. What we will do then is first make an ECG. Just as a starting point to see the result of it. If children have this more often, then we can also do a Holter, then you have to walk around with a little case, but well, for that it has occurred too infrequently and then you won’t see it. So, we will start with the ECG, and I think that it would be good to ask the pediatric cardiologist to think about this, and in this case it’s in Breda or Rotterdam, so I don’t know what is most convenient for you. I also don’t know about the waiting times.

*D* doctor, *C* child

### Facilitators for children to speak

####  Non-verbal facilitators for children to participate 

The adults took into account a longer switching pause for the child to answer questions. Pediatricians used child directed gaze and leaned forward, reaching the child’s eye level, to elicit the child’s involvement. Without these efforts, parents usually answered instead of children. Turn allocation was mostly non-verbal in teens and verbal in younger children. When children were asked their reason for encounter, parents remained silent, thus respecting the pediatrician’s speech control in favor of their child’s participation. They furthermore nodded subtly to or made eye contact with their child to encourage them staying involved, an example of which is given in Box 5.

Box 5 Example of parents facilitating child participation

**Table:** 

37.	D: And when you think about how it started and how it is now. Is the headache increasing or does it stay the same ((doctor looks at the computer))
38.	M: ((looks at child))
39.	C: ((looks at mother))
40.	M: ((nods to child))
41.	C: Uuh, I think it did not get worse, since the beginning

*D* doctor, *M* mother, *C* child, *(())* non-verbal actions

#### Verbal facilitators for children to participate

Doctors’ verbal facilitators to allocate turns to the children included calling their names to engage them when their attention had drifted, talking in the second person singular to open the dialogue with them, instead of talking about them, adjusting their tone of voice, simplifying speech language as more appropriate to the child’s age and using the same words as the child had displayed. These adjustments were especially observed with younger children. Other verbal facilitators of child participation were rephrasing difficult questions, asking further to clarify previous answers and using positive encouragements while they were speaking, an example of which is shown in Box 6.

Box 6 Example of parents stimulating child participation

**Table:** 

29.	D: And what are the moments that you think, this feels better, this is ok, the belly ache?
30.	C: Never, right? ((looks at mother))
31.	M: Sometimes it gets a little less, right?
32.	C: Well, yes, not now.
33.	M: No, not now, but the doctor is asking if it happens sometimes that you do not have as much belly ache, is there a time you have less pain?
34.	C: Yes
35.	D: When is that?
36.	M: When is the pain less?
37.	C: Nothing, I do not know
38.	M: You have the belly ache, so you must think
39.	C: ((thinks for a few seconds)) what?
40.	M: When is the bellyache getting less?
41.	C: Uhm, well not when I am eating, because that is when the belly ache gets worse
42.	M: Ok

*D* doctor, *M* mother, *C* child, *(())* non-verbal actions

#### Facilitating actions for children to participate

Pediatricians often enquired the child about his or her subjective symptoms even though the parent seemingly had taken over the conversation by providing a detailed history. Physical objects or visual score lists such as the Bristol stool score [8] or an illustrated anatomical children’s atlas [9] to explain medical concepts would easily draw the child’s attention (Box 7). Parents stimulated the participation of younger children by verifying their answer with the child, as shown in Box 8. Children promoted their own participation by instigating new subjects, adding information on the current subject or by disagreeing on answers given by their parents (Box 9). They also demanded attention by embodied actions, as shown in Box 10.

Box 7 Example of using physical objects to explain medical concepts to children

**Table:** 

323.	((child is browsing in an illustrated anatomy atlas for children)) D: Hey “child’s name”, what we will do, we will take a very special picture of your hand, not just the outside, but just like this book, so we can see the bones, so we are going to take a picture and then we see all the bones of your hand. That you will do with mom in a minute. We’ll go to the other room and there we’ll make the picture and then you are going home nicely. You have done so well.
324.	C: ok
325.	D: and I am going to figure out why you are so tall.
326.	C: ok
327.	D: And then we’ll call mom about that, yes.
328.	C: ((holds himself next to the book)) And I am taller than this book!
329.	D: You are taller than this boy, yes, I see that too, look!

*D* doctor, *C* child, *(())* non-verbal actions

Box 8 Example of parents verifying their answer with children

**Table:** 

25.	D: is it always there?
26.	M: yes, wakes up, and goes to sleep with it. ((pauses, looks at child)) I think in the beginning it wasn’t like that, hey, that you wake up with it, or did you?
27.	C: ((nods))

*D* doctor, *M* mother, *C* child, *(())* non-verbal actions

Box 9 Example of children interrupting/disagreeing with parents

**Table:** 

54.	D: Yes, and how often do you pooh on the toilet? Are you going to the toilet every day to pooh?
55.	C: Uhm
56.	M: No, we have recorded that since then.
57.	C: No, I’ve got this form, but I…
58.	D: Did you bring that form or not?
59.	C: No, 2, two times maybe, sometimes I don’t pooh for two days.
60.	D: Sometimes you don’t pooh for two days?
61.	M: No, one day you don’t pooh, and then you pooh again, I think you’ve got everytime…
62.	C: No, two days
63.	M: Yes, then you don’t pooh today, but then you do pooh tomorrow, you never have two days…
64.	C: Yesterday I also did not pooh.

*D* doctor, *M* mother, *C* child

Box 10 Example of children using actions to instigate talks

**Table:** 

472.	((child and doctor return to their seats after taking a blood pressure measurement)) D: Yes, so it’s always quite a search, when you must do something, then you must search in your head where it is, where can I find it? And the pills make your head like a cleaned-up bookshelf.
473.	C: ((rubs arm and stretches arm))
474.	M: ((takes arm of child and rubs it))
475.	D: Does your arm still hurt?
476.	C: yes ((smiles))
477.	D: A little tingling?
478.	C: No, it’s a bit, here, very severe pain.
479.	D: Where it was tight, mom will massage it nicely. But, so, if you would use the tablet then some of the important effects that I notice is that your head becomes just more organized, and, yeah, they call that structure, that you can find things better. So that it’s less chaos, which is often quite enjoyable. You know what you can also compare it with, the effect, it’s like people with spectacles.

*D* doctor, *M* mother, *C* child, *(())* non-verbal actions

### Barriers for child participation

Barriers for child participation were especially observed in younger children. When the pediatrician talked to the parent for a longer time, used difficult medical terminology, or discussed subjects outside the child’s knowledge domain, such as the family history, the child’s attention was lost quickly: they looked around or played with the toys available on the doctor’s desk. Children limited their own participation by looking at their parents for an answer, although the pediatrician had asked them something directly.

#### Evaluation of the consultation

Interviews and questionnaires were obtained after nine consultations. Children reported high overall satisfaction, indicated that the pediatrician listened to them, often asked questions to them first, and that there was space for them to speak. Some children reported that the explanation was too difficult to understand. Furthermore, a few children reported some anxiety for attending the consultation. Children older than 12 years (*n* = 4) reported that interest in the problem by the pediatricians and good explanations were important during the medical encounter.

## Discussion

The aim of this study was to assess and describe actual child participation in outpatient clinic consultations and define facilitating moments and behavior, conversation themes, and additional circumstances which could promote child participation.

Compared to previous studies, our results showed a surprisingly high child participation as measured in speech turns, consisting of 28% compared to a range of 7–11% in previous studies [5, 10, 11]. As it has been 20 years since child participation was assessed worldwide [5], clinical implementation of new medical interviewing techniques could explain the improved child participation that we measured in speech turns [2, 12]. The doctor’s participation in turns in our study was comparable to results from earlier studies, but parental turns were 15% lower. Therefore,child participation increased at the expense of parent participation. This might as well be a sign of child emancipation, or children being verbally stronger to express themselves than previously seen. Each participant promoted better child participation in the triadic interaction: Pediatricians allowed for equal turn taking during the interview and allocated their questions first to the child even if the child did not give an no immediate answer. Parents provided space for the child’s answer or verified their answer with them. Children increased their own participation by instigated talks. Cahill et al. observed similar actions that improved child participation, including the doctor’s invitation to children to speak, child-directed gaze, and giving children time to answer [13].

Based on word count, however, child participation in our study remained limited to only 11%, which was two times lower than parent participation. To the best of our knowledge, Aronsson and Rundström published the only other study that used word count as a measure for child participation [4]. They showed similar results to ours, with 8% child participation compared to 34% for parents. The limited number of words per speech turn indicates a conversation pattern with mainly short answers given by the children, in line with our qualitative observations. Furthermore, children often involved their parents to formulate answers, resulting in short answers and turn allocation to the parent, who answered more extensively. This explains increased child participation in turns with limited contribution in words.

We observed considerable differences in child participation between consultation segments. Most active child participation (as assessed by word and turn counts) occured during history taking. Thematic analysis confirmed that providing medical and psycho-social information comprised almost half of total child participation in the consultation. By contrast, the verbal participation during the decision-making segment was very limited. Qualitative analyses showed that most children were not participating actively during decision-making for multiple reasons, such as the absence of shared decision-making with child or parent, starting with the medical summary or explanation while the child was getting dressed after physical examination, lengthy explanations directed to parents and use of difficult language. These observations are in accordance with previous studies which show that, although children are involved in providing medical information, they only receive information in return to a limited extent [14–17]. Furthermore, we doubt whether the short confirmatory answers that children gave during the decision-making segment of the consultation truly indicate full understanding, ultimately representing a well-considered consent for decision-making. As participation during decision-making consisted most of the time of listening to the pediatrician, this would arguably be a more passive form of participation, which cannot be measured as word or turn counts. As toys or child-friendy medical books were able to capture the attention of the child, the use of appropriate graphics might offer a better opportunity for the child to engage in medical explanations and decision-making dialogues.

Our results showed a higher number of instigated talks by children (on average 20 per consultation), with considerable variation between participants. This is in sharp contrast with Cahill et al., who did not observe any instigated talks, and considerably higher than observed by Jenkins et al. [6, 13]. We observed many triadic conversation styles, guided by the pediatrician. This may have promoted the child’s opportunities to instigate talks. However, as seen previously, adults often reject new subjects instigated by children [18]. Therefore, children still have limited power to steer the conversation in their desired direction.

This study was limited by the small sample size for quantitative outcomes. Furthermore, participants knew they were filmed and were aware of the overall study theme. This could have influenced the pediatrician’s and parent’s eagerness to promote child participation, where children on the other hand might have felt intimidated. In contrast, previous studies used older video recordings from a large data collection or informed patient and physician without specifying the research question [5, 10, 14]. Futhermore, the inclusion of only new outpatient consultations compared to studies which also included follow-up consultations might have increased child participation outcomes. According to van Dulmen et al., new consultations results in more interaction between pediatrician and children, compared to follow-up consultations [16]. Because being anxious or shy was a reason to refuse participation, it is possible that our results are skewed by selecting children with a higher ability to participate in the medical encounter. The study group might therefore not be a representative sample of the entire population in our outpatient clinic. Only female pediatricians participated in our study, as the only male pediatrician in the department was part of the research team (CSvW) and too involved in the issue of child participation to be a representative study participant. In an earlier study, physician gender did not influence child participation [19]. Therefore, we feel it is unlikely that our selection of only female pediatricians has confounded our results [19].

Despite these limitations, this study is the first study researching child participation combining quantitative and qualitative outcomes in 20 years. We combined multiple quantitative measurements (e.g., instigated talks, participation measured in words and turns) which provided a nuanced picture of actual child participation. Furthermore, our study performed diligent verbatim analyses. Outcome measurements were defined by three researchers, and consultations were rated by three researchers to compare individual judgements and reach consensus. This, in our opinion, ensured a solid base for the current results and provided clues for the expansion of future investigations.

## Conclusion

Our study, consisting of multiple quantitative and qualitative outcomes, represents the current state of child participation in a general pediatric outpatient setting. Although child participation as measured in speech turns is much higher than recorded previously, and children instigated more talks than previously described, overall child participation in words and involvement during decision-making does not appear to have increased over time. Children are mainly involved in psychosocial issues. If pediatricians and parents maintain a triadic conversation style throughout every stage of the medical encounter, child participation may increase. Both quantitative and qualitative outcomes confirm the ability and willingness of children to participate in medical encounters, which should be respected and investigated more in the future.

### Supplementary Information

Below is the link to the electronic supplementary material.

Supplementary file1 (DOCX 357 KB)

## Data Availability

No datasets were generated or analysed during the current study.
