# Supplementary material for: Child participation during outpatient consultations: a mixed methods study
Source: Eur J Pediatr. 2024 Apr 19;183(7):3019–28. doi: 10.1007/s00431-024-05566-8 (PMC11192691; doi:10.1007/s00431-024-05566-8)

***Supplementary Figures***


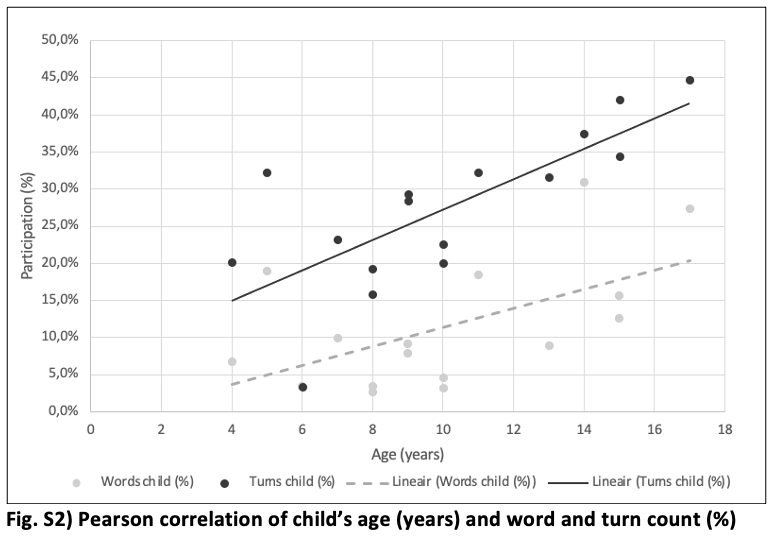
*
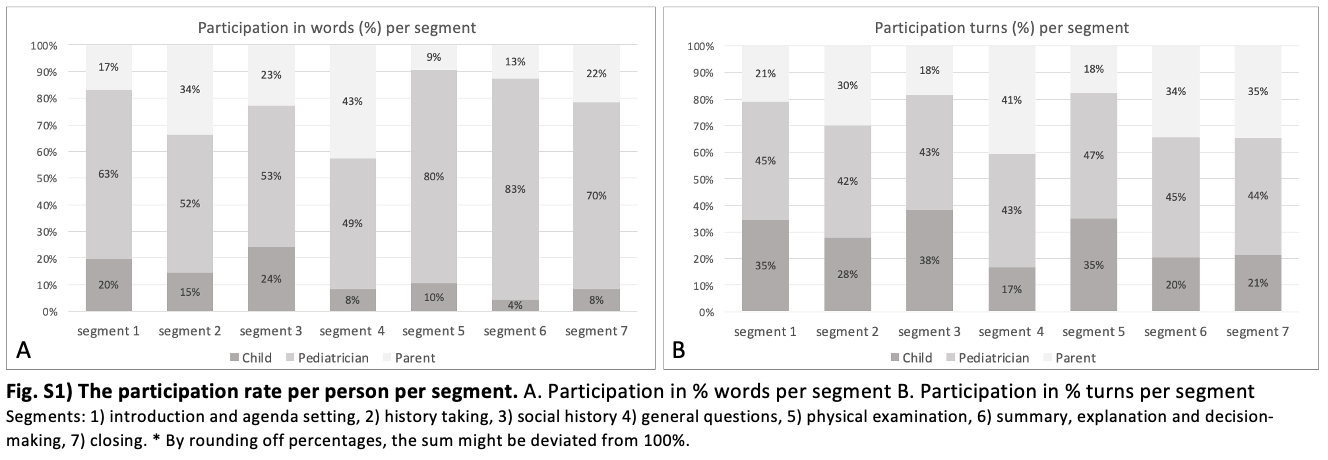
*

| Table S1. Pearson correlation | | |
| --- | --- | --- |
|  | R- value | p-value |
| Age (years) - Turn count (%) | 0,746 | < 0.001 |
| Age (years) - Word count (%) | 0,566 | 0.022 |


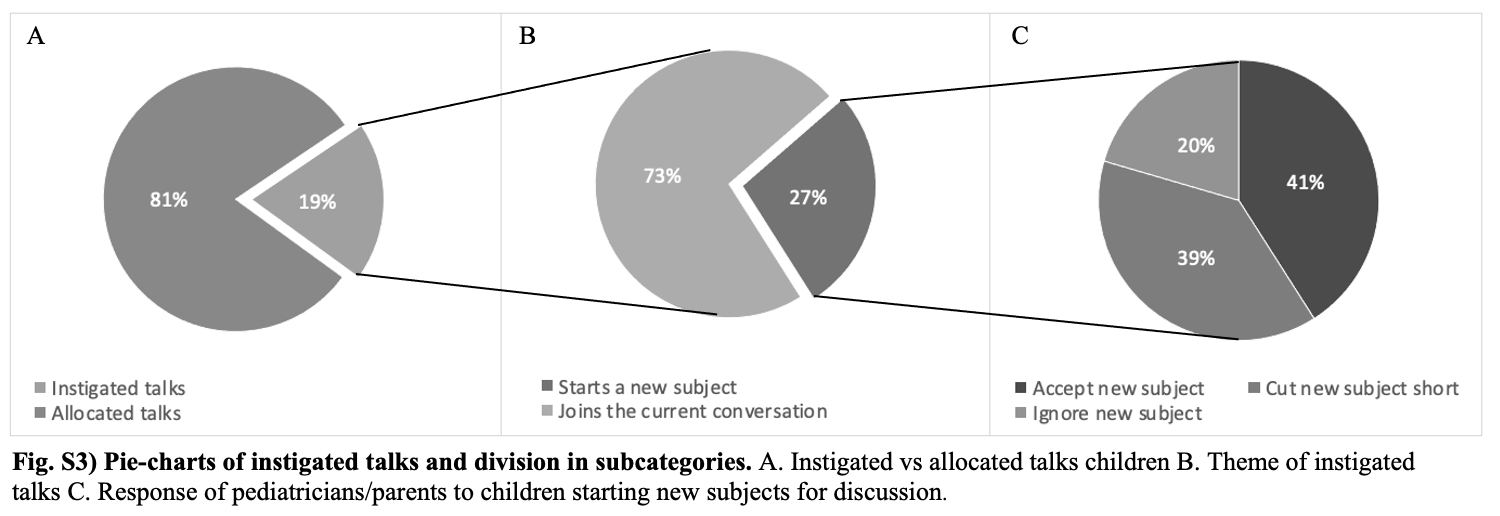

Supplement: Supplementary file 1 — Supplementary file1 (DOCX 357 KB) [file 431_2024_5566_MOESM1_ESM.docx]
